# Educational Case: Hidradenitis suppurativa

**DOI:** 10.1016/j.acpath.2026.100279

**Published:** 2026-06-25

**Authors:** Ryan C. Saal, Linnea Lackstrom Westerkam, Cristo A. Carrasco Mendoza, Paul B. Googe, Christopher J. Sayed

**Affiliations:** a Eastern Virginia Medical School, Norfolk, VA, USA; b University of North Carolina School of Medicine, Chapel Hill, NC, USA; c Kaiser Permanente Bernard J. Tyson School of Medicine, Pasadena, CA, USA; d Department of Dermatology, University of North Carolina, Chapel Hill, NC, USA

**Keywords:** Pathology competencies, Organ system pathology, Pathophysiology, Skin, Skin disease, Hidradenitis suppurativa

## Primary objective

The following fictional case is intended as a learning tool within the Pathology Competencies for Medical Education (PCME), a set of national standards for teaching pathology. These are divided into three basic competencies: Disease Mechanisms and Processes, Organ System Pathology, and Diagnostic Medicine and Therapeutic Pathology. For additional information, and a full list of learning objectives for all three competencies, see https://doi.org/10.1016/j.acpath.2023.100086.^1^

Objective SK1.1: Pathophysiology of changes in the skin. Describe the pathophysiologic basis for changes in the color, surface texture, swelling, temperature, and sensitivity of skin.

Competency 2: Organ system pathology, Topic: Skin (SK); Learning Goal 1: Classification of skin disease.

## Patient presentation

A 35-year-old woman presents to an urgent care with a chief concern of a painful abscess in her left axilla. Upon questioning, she reports a history of many similar abscesses and nodules in the past that have left behind severe scarring on her axillae, groin, and under her breasts. The lesions are severely painful and often produce bloody and purulent, malodorous discharge. She states that this has been going on since her teen years and has worsened over time resulting in several visits to the emergency room where incision and drainage has been performed. She has been given many courses of antibiotics in attempts to resolve this condition. She typically works in offices where she can limit her physical activity when lesions are painful but has to call out of work often due to the pain from her skin condition. Past medical history is significant for type 2 diabetes and depression. Her only medication is metformin. She does not consume alcohol but she currently smokes one pack of cigarettes daily and has for the last 15 years.

## Diagnostic findings, Part 1

**Fig. 1:**
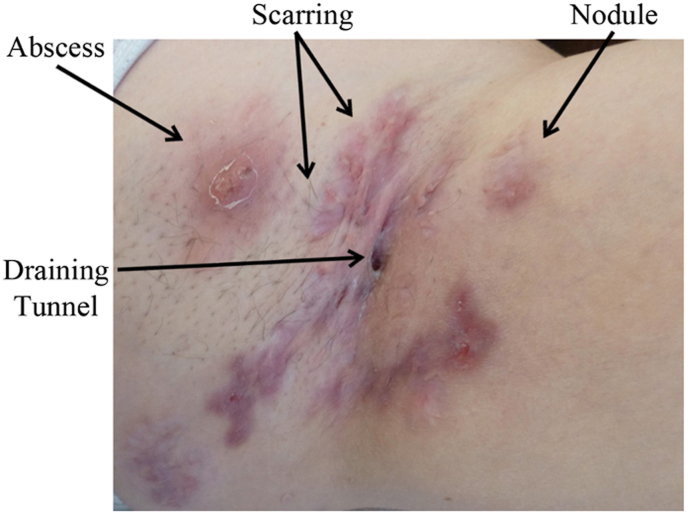
Clinical presentation. Erythematous inflammatory nodules, an abscess, hypertrophic scars, and a draining tunnel involving the inferior portion of the patient’s left axillary vault.

Vital signs are blood pressure of 125/83 mmHg, respiratory rate of 18 breaths per minute, heart rate of 87 beats per minute, and temperature of 98.9°F. Her body mass index (BMI) was 37. A fluctuant abscess is present in the left inferior aspect of the axillary vault with additional erythematous, inflammatory nodules, hypertrophic scar, and a draining tunnel (Fig. 1). Further exam reveals similar scarring and additional inflammatory nodules and draining tunnels in the right axilla, inframammary folds, and inguinal creases. Range of motion of the left arm is limited due to pain.

## Questions/discussion points, Part 1

### What is the differential diagnosis based on the clinical presentation and history?

The patient is presenting with multiple inflamed nodules, draining tunnels, and abscesses, which is suspicious for an inflammatory or infectious process. The differential diagnosis would include, but not be limited to, hidradenitis suppurativa (HS), furuncles, carbuncles, cellulitis, folliculitis, and inflamed epidermal inclusion cyst. Given the involvement of the axillae, inframammary folds, and inguinal area, presence of draining sinuses, abscesses, and scarring, an elevated BMI and failure to resolve after numerous antibiotics treatments, a diagnosis of HS is the most likely diagnosis. This patient meets clinical diagnostic criteria of 1) presence of typical lesions including inflammatory nodules, abscesses and tunnels, 2) occurring in typical intertriginous locations, and 3) two or more lesions within a 6-month period.^2^ Moreover, the chronic nature of this patient’s condition of draining sinuses, abscesses, and scarring in multiple different anatomical locations makes a diagnosis of HS more likely rather than recurrent, singular cysts, or carbuncles.

## Diagnostic findings, Part 2

**Fig. 2:**
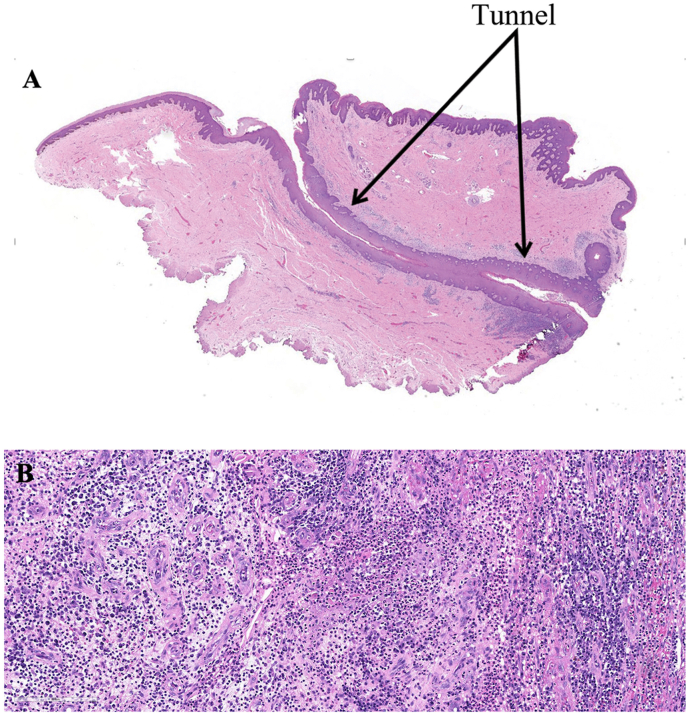
Hidradenitis suppurativa. A skin excision shows a tunnel lined by squamous epithelium that leads into the deep dermis (A, 1x, hematoxylin and eosin). Granulation tissue with neutrophils, lymphocytes, and plasma cells is present adjacent to the tunnel (B, 20x, hematoxylin and eosin).

A skin biopsy was performed with the histological features shown in Fig. 2A and B.

## Questions/discussion points, Part 2

### How would you describe the histological features observed in the biopsy? What is the diagnosis?

Though not typically necessary as part of a diagnostic workup, histologic findings classically demonstrate follicular plugging and hyperkeratosis with dense fibrosis in areas of longstanding disease. A dense lymphocytic infiltrate is seen around intact follicles while in areas of follicular rupture, there is often abscess formation with intense infiltration of neutrophils and macrophages. The tunnels may be epithelialized or lined by a scar and are typically surrounded by inflammatory cells and fibrosis. Tunnels typically are confined to the dermis, though they may extend to the subcutis, and are often filled with granulation tissue mixed with neutrophils, macrophages, and plasma cells (Fig. 2). A biopsy is not usually required to confirm the diagnosis of HS as the physician is able to diagnose this disease based on clinical presentation in addition to its corresponding disease stage. However, in some cases, a biopsy may be indicated for areas of recalcitrant or progressive ulceration, when squamous cell carcinoma is suspected, or when an alternative diagnosis such as cutaneous Crohn’s disease is being considered.^3^

### What is HS? Describe its epidemiology and potential risk factors

Hidradenitis suppurativa is a chronic inflammatory disease characterized by painful abscesses, nodules, tunnels, and scarring of intertriginous areas, such as the inframammary, groin, perianal, perineal, and axillary regions.^4^ The prevalence of HS is approximated to range from 0.1 to 1.2% and typically affects young adults.^4^ There is a higher prevalence of HS in women than in men (3:1) and Black people are three times more likely to be affected by this disease when compared to White people.^4^^,^^5^ Smoking and obesity are two potential risk factors that have been associated with HS, though causality is not clear; an increased BMI and/or smoking may result in an increase in HS severity.^6^

### What is the pathogenesis of HS?

The exact etiology of HS is unknown but is thought to include both genetic and environmental factors. Almost half of patients with HS have a family member that is also affected. Environmental factors may include overweight/obesity and cigarette smoking.^7^

Hidradenitis suppurativaassociated inflammation is thought to begin at the hair follicle with hyperkeratosis and eventual follicular rupture. Mechanical factors such as friction from clothing or superficial trauma from shaving may trigger disease activity as well. Given its pathogenesis, treatments targeted at destroying hair follicles, such as laser hair removal, may be effective.^8^^,^^9^ In addition, disruption of the gut-skin axis and dysbiosis of the cutaneous microbiome have been observed, and dysregulated bacterial response pathways may contribute to an overactive immune response in HS-affected skin.^8^^,^^10^ Moreover, exposure to the follicular contents and bacteria triggers inflammatory cascades involving local innate immune cells and the production of tumor necrosis factor-alpha (TNF-ɑ) and interleukin-1 (IL-1).^11^ These specific cytokines allow for the recruitment of innate and adaptive immune cells to the site of HS lesions, resulting in the inflammatory process, pus production, and scar formation seen with this disease.^11^ Hidradenitis suppurativa involves a cascade of other cytokines and other inflammatory pathways, such as IL-17 and JAK signaling pathways. All of these immune signaling molecules are potential targets of current therapies and drug trials for HS.^12^

### Describe the staging systems most commonly used for HS

Several staging systems for HS exist, including the Hurley Staging, the HS Physician’s Global Assessment scale (HS-PGA), the HS Clinical Response (HiSCR) score, and the International HS Severity Score System (IHS4; Table 1^13^, ^14^, ^15^).

**Table 1:** Different Hidradenitis Suppurativa Staging Systems.^13^, ^14^, ^15^

Staging System	How Staging Is Determined	What It Is Evaluating
Hurley staging	Extent of inflammatory lesions and scarring	Disease severity (3 stages)
HS-PGA	Lesion (nodules, abscesses, tunnels) count	Disease severity (6 stages)
HiSCR	Inflammatory lesion (nodule and abscess) count	Treatment response
IHS4	Lesion (inflammatory nodules, abscesses, draining sinuses) counts multiplied by 1, 2, and 4, respectively	Disease severity (3 stages)

HiSCR: HS Clinical Response score; HS-PGA: HS Physician’s Global Assessment scale; HS: hidradenitis suppurativa; IHS4: International HS Severity Score System.

**Fig. 3:**
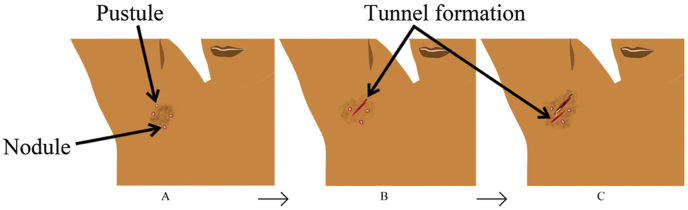
Progression of hidradenitis suppurativa lesions. Early disease typically includes nodules, pustules, and abscesses (A). Disease may progress to limited tunnel formation and scar (B). Advanced disease has more extensive tunnel formation and scar (C).

The Hurley staging system is the most commonly used staging system for HS. It was historically created to evaluate disease progression and the need for surgery in patients with HS. There are three Hurley stages: Stage I patients have recurrent nodules and abscesses but no scar formation; Hurley Stage II patients have recurrent inflammatory lesions, as well as limited tunnel formation; Hurley Stage III consists of diffuse involvement of a skin area with interconnected tunnels, scars, and inflammatory lesions (Fig. 3).^13^

The HS-PGA is another staging system that uses lesion counts, including nodules, abscesses, and sinus tracts, to assess disease severity. The HS-PGA has 6 stages, compared with Hurley staging’s 3 stages. The stages include clear, minimal, mild, moderate, severe, and very severe and are dependent on the number of lesions.^14^

The HiSCR is used to evaluate treatment response and is calculated based on the number of inflammatory lesions (nodules and abscesses) before and after treatment.^14^

The IHS4 is a validated scoring system that includes lesion counts (inflammatory nodules, abscesses, and draining sinuses). Counts are multiplied by 1, 2, and 4, respectively. The total score corresponds with disease severity: a score of less than 4 corresponds with mild, 4–10 corresponds with moderate, and greater than 10 designates severe.^15^

### What diseases are associated with hidradenitis suppurativa?

Hidradenitis suppurativa comorbidities include metabolic syndrome, type 2 diabetes mellitus, cardiovascular disease, polycystic ovarian syndrome, other inflammatory conditions (i.e. inflammatory bowel disease, such as Crohn’s disease, and inflammatory joint conditions), and mental health disorders (i.e. depression, suicide, substance use disorders). Hidradenitis suppurativa and its associated diseases have a significant impact on quality of life and patients may require an interdisciplinary team for optimal care.^16^

### What are the drug treatments for HS?

Topical antiseptics, such as chlorhexidine, povidone-iodine, chloroxylenol, isopropyl alcohol, hexachlorophene, benzalkonium chloride, and hydrogen peroxide, and topical antibiotics, such as clindamycin, are often used to manage the inflammatory component of mild HS.^17^^,^^18^ If no response occurs, then oral antibiotics (e.g. doxycycline, clindamycin, rifampicin) and intralesional steroids are added to the treatment regime to control more moderate HS.^13^^,^^19^^,^^20^ Hormonal therapies such as combination oral contraceptive pills and spironolactone may also be of benefit in mild-moderate disease as longer-term maintenance therapies or they may be used as adjuncts in more severe disease. Moderate-to-severe disease often requires aggressive long-term medical management.

Adalimumab is a human monoclonal IgG1 antibody that targets membrane-bound TNF-α and was the first Food and Drug Administration–approved biologic therapy for moderate-to-severe HS. This medication prevents TNF-α from contributing to the inflammatory pathway associated with HS and helps prevent immune cell recruitment to the site of follicular occlusion. Several studies have demonstrated the efficacy of adalimumab for the treatment of HS. However, it is important to note that the dosing schedule is increased when comparing adalimumab dosing for other indications, such as Crohn’s disease and psoriasis.^17^ Furthermore, the overall disease improvement is significantly less for HS patients when compared to patients with other diseases treated with adalimumab, such as psoriasis and rheumatoid arthritis.^21^, ^22^, ^23^ For those HS patients who do not respond to adalimumab, they may transition to alternative biologic medications in order to optimize clinical results. Infliximab, a chimeric monoclonal IgG1 anti-TNF-α antibody, is often used as a second-line biologic to treat HS as it may be more efficacious than adalimumab for severe HS treatment due to dosing flexibility (Table 2).^17^, ^18^, ^19^, ^20^, ^21^, ^22^, ^23^, ^24^

**Table 2:** Hidradenitis Suppurativa (HS) Treatments.^17^, ^18^, ^19^, ^20^, ^21^, ^22^, ^23^, ^24^

Treatment Options	
Drug or Procedure	Mechanism of action
***Medical***	
Topical antibiotics/antiseptics (i.e. clindamycin, chlorhexidine, benzoyl peroxide)	Decreases bacterial colonization to reduce immune response
Oral antibiotics (i.e. clindamycin, rifampin, doxycycline, minocycline)	
Intralesional steroids	Inhibits production of leukotrienes and inflammatory cytokines
Adalimumab	Human monoclonal IgG1 antibody that targets membrane-bound TNF-α^a^
Infliximab	Chimeric monoclonal IgG1 anti-TNF-α antibody
***Procedural***	
Hair reduction lasers (i.e. Nd:YAG^a^, alexandrite, diode, and intense-pulsed light)	Follicular reduction
Incision and drainage	Drainage of pus from an acute abscess for pain relief
Deroofing	Tissue-sparing technique to remove HS-affected tissue
Excision	Tissue removal of HS-affected area down to the subcutaneous fat

a HS: hidradenitis suppurativa; Nd:YAG: neodymium-doped yttrium aluminum garnet; TNF-α: tumor necrosis factor-alpha.

Interleukin-17 is a cytokine responsible for increasing the production of other neutrophil-attracting chemokines involved in HS inflammation.^11^ The IL-17 inhibitor, secukinumab, was recently approved for treatment of HS. Another IL-17 inhibitor, bimekizumab, is used for treatment of other inflammatory conditions and is currently in clinical trials and may be approved for HS treatment in the next few years.^25^^,^^26^ Other drug treatment options include acitretin, cyclosporine, dapsone, isotretinoin, alitretinoin, and colchicine.^17^

### What are the methods for treating HS surgically?

Procedural management for HS includes neodymium-doped yttrium aluminum garnet (Nd:YAG) lasers , deroofing procedures, and excisions. A neodymium-doped yttrium aluminum garnet laser is a 1064nm laser used for hair reduction in treated areas.^18^ Since HS inflammation involves hair follicles, decreasing the number of hair follicles present frequently reduces the number and frequency of inflammatory nodules.

Incision and drainage may be used in situations such as the aforemntioned patient encounter for acute pain relief of an abscess. While recurrence rates are high for areas where abscesses have recurred, relieving the pressure provides rapid pain relief.^18^ Wound packing generally does not improve outcomes and leads to more pain and difficulty with wound healing.^27^ Deroofing, or unroofing, procedures are tissue sparing. Using a fistula probe, the sinus tracts are defined and the overlying tissue removed.^18^ In excisional procedures, tissue in the affected body area is removed down to the subcutaneous fat.^18^ Both lesion-directed and larger regional excisions may be used to remove HS-affected skin. For deroofing, the wounds are typically left to heal by secondary intention, while excision wounds may be healed via second intention or closed using primary linear closure, skin graft, or flap.^18^

## Teaching points

• Hidradenitis suppurativa (HS) is a chronic inflammatory disease presenting as painful abscesses, nodules, sinus tracts, and scarring of intertriginous areas, such as the inframammary, groin, perianal, perineal, and axillary regions.
• On histology, HS with limited inflammation is characterized by follicular plugging and perifollicular lymphocytes, while more intense inflammation is associated with follicular rupture and a dense infiltrate of neutrophils, macrophages, and plasma cells. Tunnels may be lined with epithelium or scar and are often filled with granulation tissue and an inflammatory cell infiltrate.
• Smoking and obesity are two potential risk factors that have been associated with HS, though the majority of patients in the US are nonsmokers and patients of normal weight can be affected.
• Pathogenesis of HS is hypothesized to begin at the hair follicle with dysbiosis, an overactive inflammatory response and follicular plugging and rupture. Disruption of the gut-skin axis and dysbiosis of the microbiome also play a role in immune system dysregulation in HS-affected skin.
• The main staging system for HS is called Hurley staging: patients with Stage I have recurrent nodules and abscess but have no scar formation; Hurley Stage II patients have recurrent inflammatory lesions, as well as sinus formation; Hurley Stage III consists of diffuse involvement of a skin area with interconnected sinuses, scars, and inflammatory lesions. Hurley stage increases as extent of tunnels and scars increase.
• Hidradenitis suppurativa comorbidities include metabolic syndrome, type II diabetes mellitus, cardiovascular disease, polycystic ovarian syndrome, inflammatory bowel disease , and mental health disorders.
• Medically, topical antiseptics, oral antibiotics, and intralesional steroids are used to treat mild to moderate HS. Adalimumab and infliximab are biologics that inhibit tumor necrosis factor-alpha and are employed to treat moderate to severe HS.
• Procedural and surgical management for HS includes a neodymium-doped yttrium aluminum garnet laser, incision and drainage, deroofing procedures, and excisions.

## Funding

The article processing fee for this article was funded by an Open Access Award given by the Society of ‘67, which supports the mission of the Association for Academic Pathology to produce the next generation of outstanding investigators and educational scholars in the field of pathology. This award helps to promote the publication of high-quality original scholarship in *Academic Pathology* by authors at an early stage of academic development.

## Declaration of competing interest

The authors declare that they have no known competing financial interests or personal relationships that could have appeared to influence the work reported in this paper.
